# Trends of utilization of Complete Blood Count parameters for patient management among doctors in Azad Kashmir

**DOI:** 10.12669/pjms.36.5.1885

**Published:** 2020

**Authors:** Malik Mahmood Ahmed, Sanniya Khan Ghauri, Arslaan Javaeed, Nazish Rafique, Wajid Hussain, Nasir Khan

**Affiliations:** 1 Malik Mahmood Ahmed, MBBS, DCP, M.Phil. Azad Jammu & Kashmir Medical College, Muzaffarabad, Azad Kashmir, Pakistan; 2 Dr. Sanniya Khan Ghauri, MBBS, MRCEM. Department of Emergency Medicine, Shifa International Hospital, Islamabad, Pakistan; 3 Dr. Arslaan Javaeed, MBBS, M.Phil., MHPE, MHR. Poonch Medical College, Rawalakot, Azad Kashmir, Pakistan; 4 Nazish Rafique, BS Clinical Laboratory Sciences, Faculty of Allied Health Science, AJK University, Azad Kashmir, Pakistan; 5 Wajid Hussain, BS Clinical Laboratory Sciences, Faculty of Allied Health Science, AJK University, Azad Kashmir, Pakistan; 6 Nasir Khan, BS Clinical Laboratory Sciences, Faculty of Allied Health Science, AJK University, Azad Kashmir, Pakistan

**Keywords:** Complete blood count, Physicians preference, Utility, Pakistan

## Abstract

**Objective:**

To determine the trends of utilization of CBC parameters in patient management among doctors in different hospitals in Kashmir, Pakistan.

**Methodd:**

A self-administered questionnaire-based survey was carried out amongst doctors working in four hospitals of Kashmir i.e. Combined Military Hospital, Muzaffarabad, Combined Military Hospital, Rawalakot, Abbas Institute of Medical Sciences, Muzaffarabad, and District Hospital, Kotli during August to December 2017.

**Results:**

Out of 500 physicians, 217 physicians answered the questionnaire, representing a response rate of 43.4%. Only three of the 11 parameters in the CBC report i.e. hemoglobin, white blood cell count and platelets were selected as frequently or always useful by more than 80% of physicians. Rest of the eight parameters of the CBC were found useful by less than 80% of the physicians. Most agreed that the current format of a CBC report gives adequate information.

**Conclusion:**

The present study concludes that majority of the physicians utilize only three of the basic parameters on the complete blood count. An educational intervention can be planned for the physicians to increase their knowledge about the utility of other parameters.

## INTRODUCTION

Complete Blood Count (CBC) is defined as a blood test requested by a doctor or other medical professional which gives an information regarding the cells in the human blood such as red blood cells, platelets and white blood cells.^1^ It also gives the concentrations/relative proportion of various cellular elements. It is also known as full blood count (FBC), or full blood exam (FBE). It usually consists of eight to ten parameters including Hemoglobin (Hb), indices about red blood cells such as Red Blood Cell count (RBC), Mean Cell Volume (MCV), Mean Corpuscular Hemoglobin (MCH), Mean Corpuscular Hemoglobin Concentration (MCHC), Red cell Distribution Width (RDW), white blood cell parameters such as total White Blood Cell Count (WBC) and differential count of neutrophils, basophils, eosinophils, monocytes and lymphocytes, and Platelet count.^2^ CBC is an important hematological test used routinely in clinical decision making whenever a patient presents to a medical facility. It is the most commonly ordered test by the physicians and is used to highlight different causes of anemia, infection, various hematological cancers, states of low or high platelet count or a response to treatment.^3^ However, the utility of all the parameters in a routine CBC by the treating physician is unknown in the Pakistani population.

Various studies have shown that most physicians utilize only the four basic parameters such as Hb, hematocrit, WBC and Platelet count when looking at a CBC report.^4^,^5^ As it is the most commonly ordered test, it is imperative to have a thorough understanding of its parameters, the significance of the differential count and the frequency for which it needs to be ordered.

Since the advent of the automated hematology analyzers, the reporting of CBC has gone a major transformation.^6^ Much detailed reports regarding cell type and indices are available to the physicians. Apart from the red cell indices and the red cell morphology, the white blood count can be reported in two different ways. Previously it was based on counting hundred cells on microscope and then reporting it according to the percentage of each specific cell such as neutrophils, lymphocytes, eosinophils etc. To interpret the differential correctly, it is imperative to multiply the percentage of each specific cell with the total white cell count. On the other hand, automated hematology analyzers count hundreds of white blood cells and then report differentials based on those number.^5^ Most laboratories report both percentages and absolute cell counts but it creates confusion for the physicians to interpret. Some laboratories report only the absolute WBC, but it is still to be known if physicians are comfortable with the reporting and find it adequate. Newer studies have suggested role of platelet indices such as platelet volume, plateletcrit, platelet distribution width etc. as upcoming markers for diagnosis and prognosis.^7^ Upcoming role of reticulocytes in diagnosis of anemia such as reticulocyte hemoglobin content or reticulocyte mean volume could mean more information for the physicians to interpret.^8^,^9^ Thus, it is suggested that the information contained in the complete blood count is helpful for clinician in the management of patient, but they may not be using it effectively.^10^

Pakistan is a low middle-income nation without public healthcare system where the patient is responsible to bear the burden of the expenses incurred during treatment. Inadequate knowledge and utilization of the most common test, CBC, cannot only lead to under/over diagnosis but also unnecessary repetition.^11^ The objective of the study was to see how effectively the clinicians, utilize the information provided to them in complete blood count report and in the form of reticulocytes results.

## METHODS

A cross-sectional study was conducted by using a self-administered questionnaire. This questionnaire was designed based on information obtained from the study by Sandhu et al. in United States.^4^ The questionnaire was validated by two epidemiologists. The study duration was 5-months i.e. August 2017 to December 2017. All the doctors of Combined Military Hospital Muzaffarabad, Abbas Institute of Medical Sciences Hospital, Muzaffarabad, Combined Military Hospital, Rawalakot and District Headquarter Hospital, Kotli were included through convenience sampling. Nurses and paramedical staff were excluded from the study. The questionnaire was distributed amongst doctors in medical, surgical, pediatrics and obstetrics/gynecology outpatient clinics during office hours and was collected on the same day. The data was analyzed by statistical analysis on SPSS version 23 software. Informed consent was taken from the participants before handing the questionnaire. Approval was obtained from the institutional ethical committee with reference number PMCKT/IRB/3-17 dated 9^th^ May 2017.

## RESULTS

**Fig.1:**
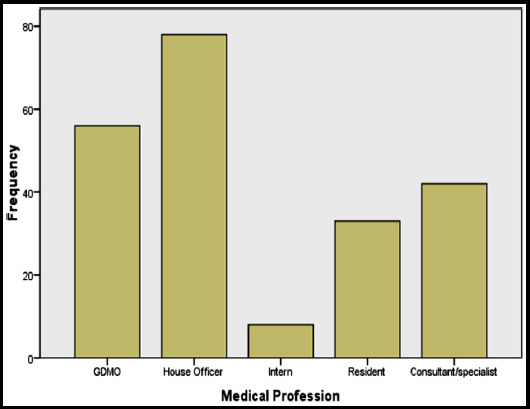
Bar chart representing the responding percentage of categories of medical profession.

A total of 500 physicians were contacted out of which 217 physicians responded to the survey (response rate 43.4%). Of the respondents, the percentage of house officers was greatest (78/217, 36%) followed by General Duty Medical Officers (GDMO) (56/217, 26%), consultant/specialists (41/217, 19%), residents (33/217, 15%) and interns (9/217, 4%). The response rate amongst physicians is shown in Fig.1. The survey was distributed amongst physicians in various departments out of which the highest response was from physicians belonging to department of surgery (29%). Response from department of pediatrics and medicine was 28% and 24% respectively whereas only 19% of the total responses were contributed by the obstetrics/gynecology department.

The self-reported utility of CBC parameters for physicians is shown in Table-I. Almost eighty percent of the physicians chose only three of the 11 parameters as frequently or always useful. Those three parameters were hemoglobin, platelet count, and WBC count. Other eight parameters of the CBC were found less than 80% useful by the physicians as shown in Table-I.

**Table-I:** Usefulness Ratings of CBC Parameters by Physicians.

Medical Profession	WBC Count		RBC Count		HGB Count		HCT		MCV		MCH		MCHC		Platelets Count		RDW Count		MPV Count			Retic Count	

	A/F	S/N	A/F	S/N	A/F	S/N	A/F	S/N	A/F	S/N	A/F	S/N	A/F	S/N	A/F	S/N	A/F	S/N		A/F	S/N	A/F	S/N
GDMO (26%)	80	20	50	50	84	16	29	71	38	62	39	61	32	68	86	14	16	84		18	82	56	44
House Officers (36%)	88	12	63	37	87	13	38	62	54	46	19	81	15	85	82	18	21	79		19	81	32	68
Intern (4%)	87	13	87	13	88	12	50	50	62	38	62	38	25	75	100	00	25	75		37	63	62	38
Resident (15%)	88	12	61	39	82	18	61	39	48	52	48	52	42	58	84	16	18	82		18	82	30	70
Consultant/ Specialist (19%)	80	20	55	45	93	07	48	52	47	53	48	52	36	64	83	17	12	88		21	79	45	55

F/A: frequently or always useful; S/N: Sometime or never useful. * Response rates are given in percentages.

Among interns and house officers, the use of mean cell volume (MCV) was higher than other physicians. 67% of the interns and 54% of the house officers were using this parameter for patient management, while other physicians were using this parameter less often. Overall, interns gave more value to different parameters of the CBC report than did other physicians. Utilization of the red cell distribution width (RDW) was low amongst all physician groups and was noted useful by an average of 18.4% of the responses. The usefulness of Red Blood Cells count was quite different among the interns and other physicians. 87% of the interns considered it always or frequently useful whereas only 50% of the GDMOs considered it useful every time as shown in Table-I. Of the respondents, more than 55% of GDMOs and interns had preferred as always useful or frequently reticulocyte count in their daily practices, while other physicians used it less than 40%.

Feedback regarding the amount of data, preferred method of WBC reporting, laboratory training required in reporting a CBC repot and preferred units for cell count is shown in Table-II. Differential in percentage was found to be more clinically useful than absolute differentials. However, 75% interns find percentage differentials more useful than absolute differentials.

**Table-II:** Feedback of physicians regarding CBC report (reported in percentages).

Medical Profession	Amount of Data			Preferred method of WBC count reporting		Laboratory Training		Preferred Units for cell count	

	Too little	Too Much	Just right	Percentage	Absolute	Adequate	Non-Adequate	/µL	/cm^3^
GDMO	2	17	81	55	45	57	43	34	66
House Officer	8	14	78	55	45	40	60	86	14
Intern	0	12	88	25	75	75	25	63	37
Resident	6	6	88	42	58	42	58	82	18
Consultant	5	17	78	62	38	48	52	60	40

Physician preferences for the reporting of cell count results are shown in Table-III. Most of the physicians preferred results to be reported in cell count/μL. In the survey we asked the physicians about the amount of data presented in the CBC report. More than 80% physicians responded that data was just right. Most of the respondents suggested that there should be no change in the CBC report.

**Table-III:** Suggestions for improvement in CBC reporting (reported in percentages).

Medical Profession	Suggestions			

	Computerized report	Peripheral film included	Signed off by hematologist	No change
GDMO	22	14	16	48
House Officer	21	8	19	52
Intern	25	37	0	38
Resident	18	9	12	61
Consultant	26	14	7	53

In our study the 52% did not want to change anything in the reporting format whereas 21% suggested that the laboratory report should be computerized (Table-III). Verification of the laboratory report by peripheral film and final reporting by hematologist was suggested by 27 percent of the responding physicians.

## DISCUSSION

This survey identified different patterns of utilization of CBC parameters as well differential counts and reticulocytes count, cell count units and report design suggestion. The response rates of 43% of physicians including GDMO, House officers, interns, Residents and consultant / specialists is much lower than our expectations. The inclusion of different category of physicians in the study gives a broader perspective regarding CBC reporting and its utility. This study showed that the use of CBC and Reticulocytes results for decision-making in patient management in Azad Kashmir hospitals is inadequate.

The study indicated that WBC count, Hemoglobin count and platelets count were the most frequently/always used by the majority of physicians. These three parameters were selected more than 80% used in the daily practices of the physician in Pakistani physicians while the study conducted by Sandhau’s in United State indicated that these parameters were used more than 90% by the physicians.^4^ Almost more than half of the physicians did not use RBC count for patient management which is similar to another study that concluded RBC count is not use commonly in decision making.^5^

In the study, MCV, MCH, MCHC have been regarding as having low utility as has been noted in other studies elsewhere.^3^,^5^ MCV has been considered as the most valuable RBC parameter in the diagnosis of anemia.^12^ In clinical practice, MCH and MCHC values are usually used by clinicians for long term follow up and are not required for acute diagnoses.

In our study RDW was found to be used much less frequently/always than the other red cell indices. In another study it has been reported that RDW was not or rarely used by around 67% of the physicians.^12^ However, it has been reported that RDW is highly under-utilized index.^13^ Our survey indicated that Mean Platelets Volume is less useful by physicians. In spite of the recent studies suggestive of clinical benefits of platelets parameters,^14^-^16^ the contribution of MPV to clinical practice was low.^17^

Most of the physicians were comfortable in using differentials as opposed to absolute numbers while reporting white blood cell count. One could argue that as it has been the norm, the physicians have an easier understanding of the differential as compared to knowing the reference ranges for absolute cell counts and converting it into percentages to understand.

Regarding units of measurement of cell count such as RBC, platelets and WBC. These parameters can be reported in cubic millimeter or per microliter. Our study showed that most of physicians prefer cell count to be reported in per microliter while study conducted in United States showed that most of the physician prefer cell count in per cubic millimeter.^4^

The use of reticulocytes count in our study is low while study conducted in Addis Ababa Hospital also showed that of reticulocytes count is used quite less often.^10^ Fifty-two percent of our participants in our study felt that the amount of data presented in CBC report is just right. In our study the 52% response rate of the physicians about laboratory report design changing was nothing. About 21% physician suggested that the laboratory report should be computerized whereas 27% suggested that the laboratory report should be verified by peripheral film and reports should be done by hematologist. A study from Canada reported the perceived low utility of morphology reporting in CBC amongst physicians and suggested to omit those morphological descriptions that are not useful clinically.^18^

## CONCLUSION

The present study concludes that majority of the physicians utilize only three of the basic parameters on the complete blood count. An educational intervention can be planned for the physicians to increase their knowledge about the utility of other parameters. Complete blood count parameters are important hematological test used routinely in clinical decision making commonly in anemia and other blood disorders. However, the use of CBC parameters in the physicians surveyed in our study is found to be inadequate. Use of Red Cell indices and Mean platelets volume is very low among the physicians. Preferred method of WBC differential is the reporting of differentials in percentages. Responsible authorities must take necessary action and develop training programs to ensure adequate use of CBC parameters in daily practice of physicians for patient management.

### Recommendations

1. Educational intervention regarding RBC indices, WBC differential count and platelet indices should be undertaken amongst doctors to promote effective utilization of CBC report
2. The information obtained in a CBC report is adequate according to half of the studied population and requires no amendment
3. Further studies can be carried out amongst medical students regarding their knowledge of CBC
4. Further department-wise stratification of study population can reveal trends of utilization amongst different specialties.

### Authors’ Contribution

**MMA, AJ, SKG** conceived, designed and did statistical analysis & editing of manuscript.

**NR, WH, NK** did data collection and manuscript writing.

**MMA** takes the responsibility and is accountable for all aspects of the work in ensuring that questions related to the accuracy or integrity of any part of the work are appropriately investigated and resolved.
